# Epigenetic and genetic variation at *SKA2* predict suicidal behavior and post-traumatic stress disorder

**DOI:** 10.1038/tp.2015.105

**Published:** 2015-08-25

**Authors:** Z Kaminsky, H C Wilcox, W W Eaton, K Van Eck, V Kilaru, T Jovanovic, T Klengel, B Bradley, E B Binder, K J Ressler, A K Smith

**Affiliations:** 1 Department of Psychiatry and Behavioral Sciences, Johns Hopkins University School of Medicine, Baltimore, MD, USA; 2 Department of Mental Health, Johns Hopkins Bloomberg School of Public Health, Baltimore, MD, USA; 3 Department of Pediatrics, Johns Hopkins University School of Medicine, Baltimore, MD, USA; 4 Department of Psychiatry and Behavioral Sciences, Emory University School of Medicine, Atlanta, GA, USA; 5 Mental Health Service Line, Department of Veterans Affairs Medical, Atlanta, GA, USA; 6 Department of Translational Research in Psychiatry, Max Planck Institute of Psychiatry, Munich, Germany; 7 Howard Hughes Medical Institute, Chevy Chase, MD, USA

## Abstract

Traumatic stress results in hypothalamic pituitary adrenal (HPA) axis abnormalities and an increased risk to both suicidal behaviors and post-traumatic stress disorder (PTSD). Previous work out of our laboratory identified *SKA2* DNA methylation associations with suicidal behavior in the blood and brain of multiple cohorts. Interaction of *SKA2* with stress predicted suicidal behavior with ~80% accuracy. SKA2 is hypothesized to reduce the ability to suppress cortisol following stress, which is of potentially high relevance in traumatized populations. Our objective was to investigate the interaction of *SKA2* and trauma exposure on HPA axis function, suicide attempt and PTSD. *SKA2* DNA methylation at Illumina HM450 probe cg13989295 was assessed for association with suicidal behavior and PTSD metrics in the context of Child Trauma Questionnaire (CTQ) scores in 421 blood and 61 saliva samples from the Grady Trauma Project (GTP) cohort. Dexamethasone suppression test (DST) data were evaluated for a subset of 209 GTP subjects. *SKA2* methylation interacted with CTQ scores to predict lifetime suicide attempt in saliva and blood with areas under the receiver operator characteristic curve (AUCs) of 0.76 and 0.73 (95% confidence interval (CI): 0.6–0.92, *P*=0.003, and CI: 0.65–0.78, *P*<0.0001) and to mediate the suppression of cortisol following DST (*β*=0.5±0.19, F=1.51, degrees of freedom (df)=12/167, *P*=0.0096). Cumulatively, the data suggest that epigenetic variation at *SKA2* mediates vulnerability to suicidal behaviors and PTSD through dysregulation of the HPA axis in response to stress.

## Introduction

Suicide represents a major public health problem, claiming over 40 000 lives per year. Suicide rates have remained stable over the past 60 years at around 10–12 per 100 000.^1^ One strategy proposed by the National Action Alliance for Suicide Prevention to reduce the rate has been to target intervention efforts toward subgroups at the greatest risk, a strategy requiring the identification of reliable biomarkers capable of identifying those at current or future risk.^2^ Previously identified risk factors implicated in suicide include biological or genetic characteristics, early-life trauma, stressful life events, impulsive aggressive traits, psychopathology, inadequate social support, access to lethal means and substance abuse.^3, 4, 5^ Recent work by our group and others has identified biomarkers at the epigenetic or gene expression level capable of predicting suicidal behavior from blood.

Previous work suggests that epigenetic alterations in the spindle and kinetochore-associated protein 2 (*SKA2*) gene may represent a promising biomarker for detecting suicidal behaviors.^6^ This study determined that the cytosine, but not the thymine, allele of rs7208505 could be methylated and that higher DNA methylation at this site predicted lower *SKA2* expression in the frontal cortex of suicide completers,^6^ along with lower levels of microRNA-301a in the cortex of depressed suicide completers.^7^ Expression of this microRNA is tied to *SKA2* expression, suggesting that this observation may be a proxy of suicide-associated *SKA2* decreases. Recently, Niculescu *et al.*^8^ demonstrated significant *SKA2* expression decreases in the peripheral blood in both individuals with high suicidal ideation as well as in suicide completers relative to controls. The same group published previously on the biomarker efficacy of various peripheral blood-based gene expression biomarkers^9^ that have also subsequently been independently replicated.^10^ Data exist to suggest that these gene systems may be linked,^6^ further implicating the possible efficacy of biomarker-based suicidal behavior prediction. An important feature of both biomarker panels is the observation of consistent associations across a broad range of suicidal behaviors including suicidal ideation, suicide attempt and suicide, suggesting that dysregulation of the gene pathways associated with these biomarkers may be an important underlying feature for the progression to increasingly severe suicidal behaviors.

SKA2 has been implicated as important for enabling glucocorticoid receptor nuclear transactivation.^11^ As a result, epigenetic variation influencing levels of *SKA2* gene expression may be important for modulating the sensitivity of the hypothalamic pituitary adrenal (HPA) axis. A small amount of data exist to suggest that *SKA2* epigenetic variation may moderate the suppression of cortisol following stress.^6^ Importantly, other factors known to influence the HPA axis such as early-life trauma exposure may interact with *SKA2* epigenetic variation to moderate risk for suicidal behaviors. In addition, epigenetic variation at *SKA2* may have relevance to other psychiatric disorders that have evidence for HPA axis system disruption such as post-traumatic stress disorder (PTSD).

In this study, we used an existing data set of DNA methylation at the *SKA2* 3'-untranslated repeat (UTR) CpG (cg13989295) in the Grady Trauma Project cohort to investigate the effects of trauma exposure on *SKA2*, suicide risk and PTSD. Below, we demonstrate the effects of trauma exposure and *SKA2* on suicide risk and discuss various confounding factors influencing suicide prediction efficacy.

## Materials and methods

### Grady Trauma Project

The subjects for this study were part of a larger investigation of genetic and environmental factors that predict the response to stressful life events in a predominantly African American, urban population of low socioeconomic status.^12, 13, 14^ Research participants are approached in the waiting rooms of primary care clinics of a large, public hospital while either waiting for their medical appointments or while waiting with others who were scheduled for medical appointments. After the subjects provided written informed consent, they participated in a verbal interview and blood draw. This cohort is characterized by high rates of interpersonal violence and psychosocial stress; the majority of subjects report at least one major trauma during their lifetime, and the number of traumatic experiences in childhood and adulthood predict psychiatric symptom severity in adulthood.^14, 15^ DNA methylation analyses were performed in *N*=421 subjects from the blood of whom a subset of *N*=61 samples were also collected and analyzed from saliva.

### Johns Hopkins Center for Prevention Research Study

Data are from a prospective study conducted in a predominantly African American, urban population.^16, 17, 18^ Details of the trial are available elsewhere.^16, 17^

In brief, the trial recruited two successive cohorts of students (1196 from Cohort 1 in 1985 and 1115 from Cohort 2 in 1986) as they entered first grade in 19 elementary schools in Baltimore, MD, USA (49.8% male and 67.1% ethnic minority consistent with the population in Baltimore City schools). Since 1985, participants have been assessed through middle school, twice in young adulthood and most recently when participants were 30–32 years old. DNA methylation analyses were generated as reported previously^6^ and were restricted to the 326 individuals participating at the age of 30–32 data collection wave who at the time of this analysis provided a blood sample (60% female and 76% African American, lacking another 12 who provided blood later).

All participants provided informed consent to participate. All procedures were approved by the Institutional Review Board of Emory University School of Medicine and the Grady Health Systems Research Oversight Committee and by the Institutional Review Board at Johns Hopkins University, respectively. Samples were randomized and investigators were blinded to the phenotypic status during experimental data processing as reported previously. Detailed information on study sample characteristics and phenotype metrics for suicidal behavior, PTSD and trauma metrics appear in Supplementary Method S1 and Supplementary Table S1.

### Biological samples

For both Grady Trauma Project (GTP) and Prevention Research Study (PRC), whole blood was collected in ethylenediaminetetraacetic acid for genetic testing. As part of the GTP screen, saliva samples were also collected.

### rs7208505 DNA methylation and genotype

*SKA2* 3'-UTR DNA methylation levels were determined using normalized beta values for the cg13989295 probe from the Illumina (San Diego, CA, USA) HumanMethylation450 BeadChip from data generated previously^19, 20^ in the GTP cohort (Supplementary Method S2 and Supplementary Figure S1). In the PRC cohort, *SKA2* 3'-UTR DNA methylation levels were determined by pyrosequencing and rs7208505 genotype values were determined using reverse transcription quantitative PCR as reported previously.^6^

### Dexamethasone suppression test

In the GTP cohort, whole blood was collected under fasting conditions between 0800 and 0900 hours for baseline (that is, day 1) serum cortisol measurements. A subset of 213 subjects received a low-dose dexamethasone suppression test (DST) in which they took 0.5 mg dexamethasone orally at 2300 hours, and blood was collected on the next day (that is, day 2) between 0800 and 0900 hours. Serum cortisol at both time points was measured using a commercial radioimmunoassay kit (Diagnostic Systems Laboratories, Webster, TX, USA).

### Statistical analysis

Unless otherwise stated, reported statistics derive from linear regression analysis, adjusted for age, sex and race generated in R (http://www.r-project.org/) using the function lm (dependent variable~(cg13989295 beta value±rs7208505 genotype) × trauma metric+age+sex+race) where the dependent variable was current suicidal ideation, lifetime suicide attempt or the natural log of the day-2 cortisol values from the DST. Unless otherwise stated, the trauma metric for the GTP cohort was the total Child Trauma Questionnaire (CTQ) score, whereas the first Eigen vector of a principle components analysis combining reported sexual abuse and the mean frequency of emotional or physical abuse was used for the PRC cohort. Relevant additional covariates were determined according to the strategy presented in the Supplementary Methods (Supplementary Method S3, Supplementary Table S2). Using the Anderson–Darling test, all data distributions that rejected the null hypothesis of normality were subsequently evaluated with nonparametric tests. All statistical tests were two-tailed; *P*⩽0.05 denotes statistical significance and ± denotes the s.e.m. Where specified, genotype correction of *SKA2* 3'-UTR DNA methylation values was achieved by taking the residuals of a linear model of cg13989295 probe beta values as a function of the rs7208505 genotype. In a similar manner and as justified in Supplementary Methods S3, we adjusted *SKA2* DNA methylation levels for past history of substance abuse in all receiver operator characteristic curve analyses as the availability of different substance abuse variables in the training data set precluded the ability to account for substance-related decreases on *SKA2* DNA methylation.

Sliding window analyses were performed for visualization purposes, whereby subsamples were grouped such that all individuals falling within ±15 units for the CTQ total or ±5 units for CTQ emotional, sexual or physical abuse scores were included in the analysis. Differences in sliding window lengths allow for inclusion of similar sample numbers per group (mean sample size ~57 per window for all analyses).

## Results

### Application of suicide prediction model to the GTP cohort

We aimed to predict lifetime suicide attempt using only *SKA2* epigenetic and genetic variation without interacting covariates in order to assess the biomarker efficacy of the model independent of factors that may be independently associated with suicide. We assessed the model efficacy in both *N*=67 current suicidal ideators compared with *N*=337 controls and *N*=99 lifetime suicide attempt cases relative to *N*=321 controls. We observed poor predictive accuracy using the *SKA2*-only model that was significant for suicide attempt but not suicidal ideation (area under the receiver operator characteristic curve (AUC) SI: 0.55, 95% confidence interval (CI): 0.48–0.62, permuted *P*=0.15, AUC suicidal attempt (SA): 0.58, 95% CI: 0.52–0.64, permuted *P*=0.017). Estimation of and adjustment for individual cellular proportions did not substantially change the results of this analysis (data not shown).

### Identification of trauma interaction in the GTP cohort

Our previously published model demonstrated that *SKA2* 3'-UTR DNA methylation significantly interacted with anxiety to moderate suicidal behavior. In the GTP cohort, the total anxiety score (HAM-A) did not significantly interact with *SKA2* DNA methylation to moderate suicide attempt (interaction *β*=0.46±0.095, F=5.68, degrees of freedom (df)=13/338, *P*=0.63); however, anxiety was independently associated with both the child (*β*=0.13±0.025, F=5.13, df=1/347, *P*=3.5 × 10^−7^) and lifetime trauma scores (*β*=0.81±0.14, F=32, df=1/345, *P*=3.3 × 10^−8^). Childhood trauma scores were more significantly associated with lifetime suicide attempt (*β*=0.0083±0.001, F=59.8, df=1/410, *P*=8.2 × 10^−14^) than were lifetime trauma scores (*β*=0.0304±0.0066, F=21.5, df=1/407, *P*=4.7 × 10^-6^).

**Figure 1:**
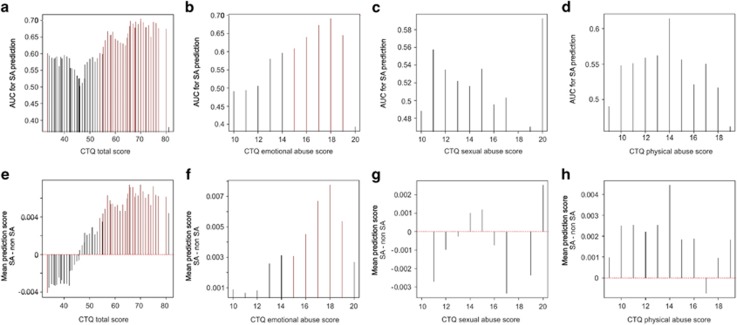
Sliding window analysis of lifetime suicide attempt prediction. Barplots of the area under the receiver operator characteristic curve (AUC) generated using the suicide prediction model (*y* axis) as a function of childhood trauma scores. For each group (*x* axis), individuals are included if they fall within a window of (**a**) ±30 points on the total Child Trauma Questionnaire (CTQ) scores, (**b**) ±5 points on the emotional abuse subscale, (**c**) ±5 points on the sexual abuse subscale and (**d**) ±5 points on the physical abuse subscale from the Grady Trauma Project (GTP) cohort. Differences in sliding window lengths (±30 versus ±5) allow for inclusion of similar sample numbers per group (mean sample size ~57 per window for all analyses). Vertical red bars represent the windows where 95% confidence intervals for the AUC do not encompass a null prediction of 0.5. Barplots of the mean suicide attempt (SA) minus non-SA score generated by the suicide prediction model (*y* axis) as a function of the middle position of sliding window encompassing all individuals within a window of (**e**) ±30 points on the total CTQ scores and those representing only ±5 points on the (**f**) emotional abuse, (**g**) sexual abuse and (**h**) physical abuse subscales (*x* axis) from the GTP cohort. All vertical red bars represent those windows where 95% confidence intervals for the AUC do not encompass a null prediction of 0.5.

**Figure 2:**
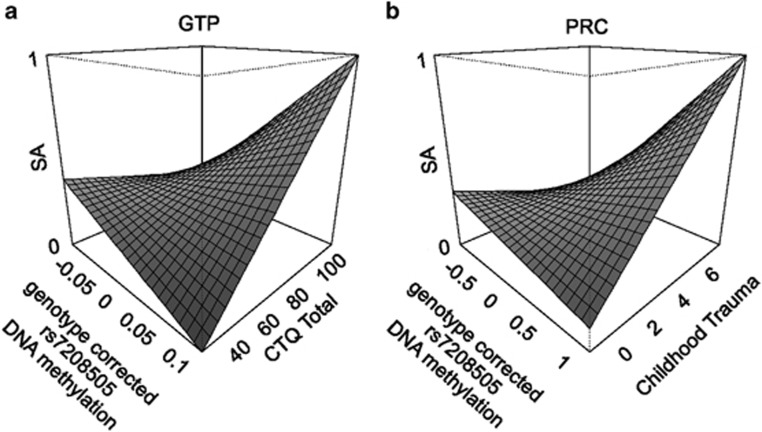
Suicidal behavior prediction models incorporating trauma exposure. A three-dimensional depiction of the effect of the genotype-corrected *SKA2* 3'-untranslated repeat (UTR) DNA methylation (*z* axis) interaction with trauma status (*x* axis) on suicide attempt as simulated in the (**a**) Grady Trauma Project (GTP) and (**b**) Prevention Research Study (PRC) cohorts (*y* axis).

We attempted to predict lifetime suicide attempt using only *SKA2* epigenetic and genetic variation without interacting covariates in subsets of individuals with different levels of child trauma exposure. We performed a sliding window analysis, generating an AUC value for suicide attempt prediction for all individuals within a range of 30 points on the CTQ. The results depicted in Figure 1a demonstrate two peaks of maximum predictive accuracy corresponding to groups in both the high and low trauma categories. Importantly, the direction of suicide attempt prediction in both cases appears to be reversed between these low and high trauma-exposed groupings (Figure 1e). This, in effect, cancels out the predictive efficacy of the *SKA2*-only model and suggests that *SKA2* 3'UTR DNA methylation may interact with the trauma status to moderate suicide risk. Linear regression modeling in the GTP cohort confirmed a significant interaction between CTQ total trauma scores and *SKA2* DNA methylation model terms after controlling for age, sex, race and lifetime substance abuse history (Table 1, Figure 2a). In the highly traumatized group, the maximum predictive efficacy of *N*=28 suicide attempt cases from *N*=37 non-attempters was an AUC of 0.71 (95% CI: 0.58–0.83, permuted *P*=0.002).

Emotional abuse, more so than physical or sexual abuse, accounted for a majority of the total CTQ score effect on suicide attempt model predictability (Figure 1, Supplementary Result S1). We next assessed model performance separately in subjects previously classified as having experienced either low or severe emotional abuse. In the severely abused group, the *SKA2* epigenetic and genetic variation model predicted lifetime suicide attempt from *N*=51 cases compared with *N*=55 non-suicide attempters with an AUC of 0.695 (95% CI: 0.59–0.8, permuted *P*=0.005), whereas stronger associations were observed in individuals having experienced emotional but not physical or sexual abuse (Supplementary Result S2). In the low emotional abuse-reporting group, *N*=47 suicide attempters were not significantly predicted, generating an AUC of 0.56 (95% CI: 0.47–0.65, permuted *P*=0.23).

### Replication of the interaction between SKA2 and trauma on suicidal behaviors

To corroborate the association of altered directionality of suicide ideation/attempt prediction in low versus high trauma-exposed subjects, we returned to the PRC cohort and assessed the direction of suicidal behavior prediction as a function of trauma exposure. We observed significant interactions for *SKA2* 3'-UTR DNA methylation and rs7208505 genotype for suicidal ideation and suicide attempt (Table 1, Figure 2b). The strength of the interaction between trauma and *SKA2* DNA methylation was strongest when modeling trauma resulting from emotional abuse as compared with physical or sexual abuse (Supplementary Table S3).

### Incorporation of trauma into the suicide prediction model

**Figure 3:**
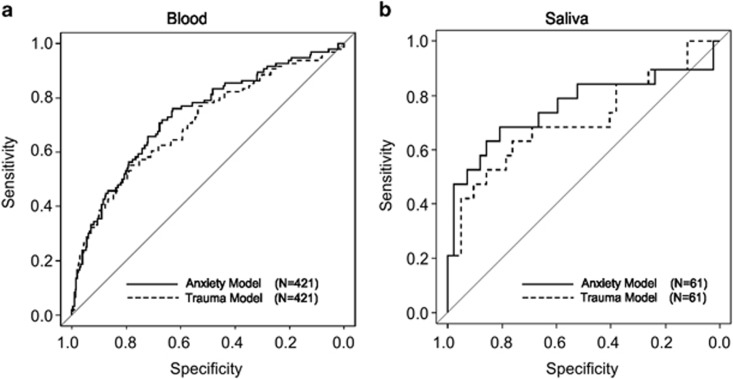
Receiver operator characteristic (ROC) curves of suicide attempt prediction in blood and saliva. ROC curves generated by the model generated in the Prevention Research Study (PRC) cohort and predicting suicide attempt in the GTP cohort in (**a**) blood and (**b**) saliva. The training set data from the PRC cohort was generated by a linear model of suicide attempt as a function of the interaction of *SKA2* 3'-UTR DNA methylation and genotype at rs7208505 with trauma scores, additively controlling for race, sex and age. Prediction in the GTP cohort input *SKA2* 3'-UTR DNA methylation adjusted for past substance abuse and rs7208505 genotype interacting with either total CTQ scores or anxiety (HAM-A) scores, whereas additively controlling for age and sex. CTQ, Child Trauma Questionnaire; GTP, Grady Trauma Project; UTR, untranslated repeat.

In light of the identified interaction of early-life trauma on suicide attempt risk, we rebuilt the statistical model from the PRC cohort, modeling the interaction of *SKA2* DNA methylation and rs7208505 genotype interacting with trauma scores, adjusting for age and sex. We assessed the efficacy in both *N*=67 current suicidal ideators compared with *N*=337 controls and *N*=99 lifetime suicide attempt cases relative to *N*=321 controls, incorporating CTQ scores as the interactive covariate. Independent validation of the model in the GTP cohort predicted current SI and lifetime SA with AUCs of 0.71 and 0.73 (95% CI: 0.65–0.78, permuted *P*<0.0001 and CI: 0.67–0.79, permuted *P*<0.0001, respectively; Figure 3a). Importantly, not adjusting *SKA2* DNA methylation for substance abuse generates very similar AUCs of 0.72 and 0.72 (95% CI: 0.65–0.78, permuted *P*<0.0001 and CI: 0.66–0.78, permuted *P*<0.0001, respectively). By comparison, the predictive efficacy of past substance abuse alone at predicting suicidal ideation (SI) and SA was AUC, 0.65 and AUC, 0.67 (95% CI: 0.59–0.72, permuted *P*<0.0001, and CI: 0.62–0.73, permuted *P*<0.0001, respectively). These results generated by *SKA2* interacting with trauma were very similar to those generated using anxiety (HAM-A) symptoms as the interactive covariate, generating AUCs of 0.70 and 0.70 for SI and SA (95% CI: 0.61–0.78, permuted *P*<0.0001 and CI: 0.64–0.77, permuted *P*<0.0001), respectively.

### Prediction using DNA from saliva

For a subset of *N*=61 individuals (Supplementary Table S1) from the GTP cohort, DNA methylation values generated from saliva DNA were available. A significant correlation was observed between blood- and saliva-derived *SKA2* 3'-UTR DNA methylation (*R*=0.96, *P*=2.2 × 10^−16^), suggesting that DNA obtained from salivary DNA may be efficacious for suicide behavior prediction.

We assessed the predictive efficacy of the PRC-generated model for prediction of suicidal behavior in GTP saliva samples. The AUC generated for the *N*=19 suicide attempters from *N*=42 non-attempters interacting *SKA2* variation with childhood abuse scores was similar to that observed in the blood at 0.76 (95% CI: 0.6–0.92, permuted *P*=0.003), whereas the AUC generated interacting *SKA2* with anxiety scores was 0.69 (95% CI: 0.53–0.86, permuted *P*=0.041; Figure 3b). Similarly to the blood-derived data, suicidal ideation with both childhood abuse and anxiety-interacting models generated AUCs of 0.66 and 0.67 (95% CI: 0.5–0.83, permuted *P*=0.14 and 95% CI: 0.49–0.83, permuted *P*=0.11, respectively).

### *SKA2* interacts with childhood trauma to predict cortisol suppression following dexamethasone treatment

DNA methylation values for *SKA2* were obtained on day 1 of a 2-day DST conducted in the GTP cohort. *SKA2* 3'-UTR DNA methylation interacted with CTQ scores to mediate the degree to which cortisol was suppressed on day 2 following the DST (Table 1, Supplementary Figure S2); however, CTQ scores alone were not associated with day-2 cortisol levels (*β*=0.0036±0.0038, F=0.88, df=1/203, *P*=0.35). Together, the data demonstrate a functional role of *SKA2* DNA methylation in mediating HPA axis sensitivity. In this way, a combination of high *SKA2* DNA methylation in traumatized individuals is associated with lower suppression of cortisol under stressful conditions.

### Application of suicide prediction model to PTSD in GTP

Epigenetic variation at *SKA2* may be efficacious for predicting PTSD, a trauma-induced disorder with HPA axis abnormalities. We therefore assessed the ability of the suicide prediction model to identify PTSD cases from the GTP cohort. Without accounting for childhood trauma, the model generated an AUC of 0.55 (95% CI: 0.48–0.63, permuted *P*=0.24) to identify the 78 PTSD cases from 203 controls. Genotype-adjusted DNA methylation of cg13989295 was not associated with PTSD; however, there was correlation with methylation of other *SKA2* CpG sites, particularly in the promoter (Supplementary Result S3, Supplementary Table S4, Supplementary Table S5). Incorporation of CTQ scores into the model generated an AUC of 0.72 (95% CI: 0.65–0.79, permuted *P*<0.0001). Consistent with the literature, PTSD demonstrated a main effect of decreasing day-2 cortisol following the DST (*β*=−1.34±0.58, F=2.17, df=3/121, *P*=0.021); however, CTQ levels significantly interacted with the PTSD status to increase post-DST day-2 cortisol levels (*β*=0.023±0.011, F=2.17, df=3/121, *P*=0.047). Notably, CTQ scores were lower among individuals with PTSD and no suicide attempt compared with those with both (Wilcoxon Rank Sum: PTSD Yes, SA No: *N*=41, mean=51±20, PTSD Yes, SA Yes: *N*=37, mean=61±24, *P*=0.095) and higher among suicide attempters without comorbid PTSD (Wilcoxon Rank Sum: PTSD No, SA No: *N*=171, mean=38±14, PTSD No, SA Yes: *N*=32, mean=53±19, *P*=3.5 × 10^−5^). There was a significant overrepresentation of suicide attempters among PTSD cases (observed probability=0.47, expected probability=0.38, *P*=0.032). Cumulatively, the data suggest the different direction of *SKA2*-mediated effects on post-DST cortisol levels with CTQ scores on day 2 may be mediated by the opposing direction of PTSD and suicidal behavior on HPA axis sensitivity.

## Discussion

In our previous work, we reported a relatively high predictive accuracy of the *SKA2* suicide prediction model across two cohorts and identified an association between genotype-corrected DNA methylation of the *SKA2* 3'-UTR and neuronal *SKA2* expression.^6^ This study expands upon these previous findings by assessing not only the predictive accuracy of the biomarker model in an independent and larger cohort but also the effect of the biomarker model independent of interacting covariates and detailing its performance in light of suicide risk factors such as childhood trauma. The AUC values reported above are moderate. There are a number of potential explanations for the lack of strength of the reported AUCs. First, the GTP cohort represents a primarily African American cohort, similar to the PRC cohort, with ~75% African Americans. As reported previously, the allele frequency of C-containing alleles is much smaller in this population relative to Caucasian, Asian and Native American individuals, suggesting that there may be a lower amount of biologically informative alleles capable of conferring DNA methylation information. Additional replication studies will be required in larger cohorts with more ethnic diversity to better understand the predictive efficacy of *SKA2* in the general population. In addition, although our supplementary analysis did not demonstrate a confounding effect of other psychiatric illnesses, it remains possible that variation is induced by different underlying psychiatric conditions as well as different subtypes of suicidality.

Second, the predictions result from retrospective data, such that epigenetic drift over time and the confounding influence of various suicide- and trauma-associated lifestyle factors may influence the prediction. In the GTP cohort, prediction of suicide attempt metrics performed stronger than predicting suicidal ideation. Our previous data indicated that elevated *SKA2* levels may be indicative of increasing severity of suicidal behaviors, which is consistent with this observation. An increased signal may be more important in a retrospective sample such as the GTP, where biological samples were taken long after a suicide attempt and factors affecting DNA methylation at *SKA2* may have caused a drift in suicide-relevant signal.

Suicidal ideation, attempt, anxiety, trauma and substance abuse metrics were obtained through different scales in the GTP and PRC cohorts. Although the results in both cohorts were consistent, each has distinct clinical features that influence *SKA2* methylation. This fact calls into question whether *SKA2* is capable of measuring any suicide-relevant biology. In light of the findings detailing that the *SKA2* epigenetic and genetic variation independent of interacting covariates was capable of similar predictive accuracies in the severe trauma cases stands as a proof of principle that *SKA2* alone may act as an efficacious biomarker in certain populations, such as highly traumatized individuals.

Initial results in a small subset of DNA obtained from saliva demonstrated a similar predictive efficacy to that observed in blood. Approximately 74% of cells in the saliva are white blood cells;^21^ therefore, a high overlap between blood- and saliva-based findings is expected. It has been demonstrated that DNA derived from the saliva may be a better proxy for the epigenetic status of the brain,^22, 23^ possibly because buccal tissue is derived from the same primary germ layer as the brain, the ectoderm. However, the relevance of peripheral biomarker signals at *SKA2* to the brain have been demonstrated previously^6^ and may result from a tissue nonspecific reprogramming of the epigenome. The implication of these observations is that salivary DNA may represent a useful collection tissue for biomarker testing, an option that would ultimately enable a less invasive and more cost-effective means to perform biomarker testing.

Our previously published model demonstrated that *SKA2* 3'-UTR DNA methylation significantly interacted with anxiety to moderate suicidal behavior that was not replicated in the GTP cohort. Although it is possible that our previously published anxiety results may be linked to underlying trauma exposure, this conclusion is not supported by the data. Instead, it is likely that the underlying factor resulting in significant interactions with *SKA2* is differential HPA axis sensitivity, which is an underlying feature of both anxiety and trauma.

We identified a significant interaction between *SKA2* variation and trauma at mediating the response to the DST, a metric of HPA axis sensitivity often dysregulated in suicidal individuals.^3, 24, 25^ Given the implicated role of SKA2 in facilitating glucocorticoid receptor nuclear transactivation and anticorrelated relationship with gene expression,^6, 8^ the observed direction of association is consistent with our previously proposed interpretation that epigenetically driven decreases in *SKA2* may inhibit the ability of glucocorticoid receptor to properly suppress natural stress response. This finding has relevance to other psychiatric disorders such as PTSD, which may have altered HPA axis sensitivity. The observed interaction is similar to that reported for other HPA axis-relevant genes including *CRHR1* and *FKBP5*. In both cases, high CTQ scores moderate the relationship between genetic variation and psychiatric symptoms or HPA–axis function.^26, 27^ Indeed, *FKBP5* has also been associated with depression, anxiety and PTSD.^28, 29, 30, 31^ Such interactions with childhood maltreatment, including those observed for *SKA2*, may result from a differential priming of the HPA axis by early-life trauma. Similar to *FKBP5*, epigenetic alterations at *SKA2* may adapt over time in the presence of heightened HPA axis sensitivity causing differential effects on the glucocorticoid receptor-negative feedback system dependent on the context of early-life exposure to stress and potentially mediated by the genetic and epigenetic context of relevant genes. These differential effects may predispose to stress-related disorders such as suicide and PTSD, which have been demonstrated to have opposing actions on the HPA axis, resulting in faster and slower clearing of post-stress cortisol, respectively. This interpretation is supported by the data as individuals with PTSD and no suicidal behaviors had generally lower CTQ scores compared with those with suicidal behavior. Thus, the observed interaction on HPA axis sensitivity may be a result of the differential contributions of these overlapping phenotypes in the subjects tested.

We observed that the *SKA2* epigenetic and genetic biomarker predicted civilian PTSD cases when child abuse was incorporated. The degree to which our observations are based on comorbid phenotypes or substance use cannot be distinguished because of the observed significant association between trauma exposure, substance abuse, PTSD and suicidality. Further work will be necessary to distinguish the degree to which *SKA2* is specific to suicide biology or more broadly affects other HPA axis-associated mental disorders such as PTSD. Future work in longitudinally collected samples will enable a robust way to test these hypotheses and to fully discern the cause versus effect nature of the identified associations of *SKA2* with suicidal behaviors.

## Figures and Tables

**Table 1:** Interactive effects on suicide phenotypes

*Sample*	*PRC suicidal ideation (*N=*325)*			*PRC suicide attempt (*N=*325)*		
*Model terms*	β-*value*	*Error*	P*-value*	β-*value*	*Error*	*P-value*
DNAm	0.002	0.001	0.19	0.000	0.001	0.84
C/T	−0.020	0.064	0.75	0.036	0.055	0.51
C/C	−0.17	0.11	0.11	−0.091	0.094	0.33
Trauma	0.062	0.022	0.004	0.034	0.019	0.07
Age	0.003	0.010	0.74	−0.007	0.009	0.40
Sex	−0.011	0.052	0.83	0.001	0.045	0.98
Past substance abuse	0.10	0.046	0.027	0.14	0.040	0.0004
DNAm × trauma	0.002	0.001	0.020	0.002	0.001	0.042
C/T × trauma	−0.053	0.045	0.24	−0.083	0.039	0.035
C/C × trauma	−0.19	0.081	0.019	−0.15	0.070	0.031
F	5			2.98		
DF	12/257			12/257		
Model *R*^2^	0.19		2.22 × 10^−7^	0.12		0.00066

	*GTP suicide attempt (*N=*421)*			*GTP post-DST Cort (*N=*209)*		
DNAm	−2.815	2.057	0.172	−14.61	7.70	0.059
C/T	1.245	1.010	0.219	7.45	3.80	0.051
C/C	2.200	1.692	0.194	11.20	6.36	0.08
Trauma	−0.003	0.004	0.549	−0.029	0.016	0.084
Age	0.000	0.002	0.938	0.003	0.006	0.643
Sex	0.135	0.042	0.001	−0.095	0.15	0.52
Past substance abuse	0.259	0.042	2 × 10^-9^	0.11	0.16	0.46
DNAm × trauma	0.100	0.048	0.037^a^	0.418	0.192	0.031^a^
C/T × trauma	−0.046	0.023	0.050^a^	−0.212	0.095	0.026^a^
C/C × trauma	−0.077	0.039	0.049^a^	−0.31	0.16	0.056^a^
F	9.92			0.80		
DF	13/393			12/188		
Model *R*^2^	0.25		2 × 10^−16^	0.048		0.64

Abbreviations: DST, dexamethasone suppression test; GTP, Grady Trauma Project; PRC, Prevention Research Study.

a Estimation of and adjustment for individual cellular proportions in the GTP cohort where these metrics were available did not substantially change the results of this analysis (data not shown).

## Notes

ZK is an inventor on patent applications for DNA methylation biomarker-based prediction of PPD, suicide and PTSD. ZK received consultant fees from Janssen Research and Development, LLC. BB receives grant support or has received awards from AFSP and the American Psychoanalytic Association Psychoanalytic Research Fund. KJR has received funding from the Burroughs Wellcome Fund, NIH, and he has an unrelated role as the cofounder of Extinction Pharmaceuticals for development of *N*-methyl-D-aspartate-based therapeutics. EBB receives grant support from the 7th framework program of the European Community (ERC starting grant, the German Federal Ministry of Education and Research, ERANet-Neuron and NIH. EBB is inventor on patent applications related to using *FKBP5* and *ABCB1* genotypes in the prediction of antidepressant response and genetic predictors of treatment emergent suicidal ideation. AKS receives or has received research support from the American Foundation for Suicide Prevention, Schering Plough Pharmaceuticals, NARSAD, the Conquer Cancer Foundation and NIH. The remaining authors declare no conflict of interest.

## References

[bib1] Centers for Disease Control and Prevention, National Centers for Injury Prevention and Control: Web-Based Injury Statistics Query and Reporting System (WISQARS) 2013 . www.cdc.gov/ncipc/wisqars .

[bib2] Force NAAfSPRPT A Prioritized Research Agenda for Suicide Prevention: An Action Plan to Save Lives Rockville, MD, USA: National Institute of Mental Health and the Research Prioritization Task Force 2014

[bib3] Mann JJ Arango VA Avenevoli S Brent DA Champagne FA Clayton P Candidate endophenotypes for genetic studies of suicidal behavior Biol Psychiatry 2009 65 556 563 10.1016/j.biopsych.2008.11.021 PMC3271953 19201395

[bib4] McGirr A Turecki G The relationship of impulsive aggressiveness to suicidality and other depression-linked behaviors Curr Psychiatry Rep 2007 9 460 466 18221625 10.1007/s11920-007-0062-2

[bib5] Shaffer D Craft L Methods of adolescent suicide prevention J Clin Psychiatry 1999 60 70 74 10073391

[bib6] Guintivano J Brown T Newcomer A Jones M Cox O Maher BS Identification and replication of a combined epigenetic and genetic biomarker predicting suicide and suicidal behaviors Am J Psychiatry 2014 171 1287 1296 25073599 10.1176/appi.ajp.2014.14010008 PMC7081376

[bib7] Smalheiser NR Lugli G Rizavi HS Torvik VI Turecki G Dwivedi Y MicroRNA expression is down-regulated and reorganized in prefrontal cortex of depressed suicide subjects PLoS ONE 2012 7 e33201 22427989 10.1371/journal.pone.0033201 PMC3302855

[bib8] Niculescu AB Levey D Le-Niculescu H Niculescu E Kurian SM Salomon D Psychiatric blood biomarkers: avoiding jumping to premature negative or positive conclusions Mol Psychiatry 2015 20 286 288 25582618 10.1038/mp.2014.180 PMC4357859

[bib9] Le-Niculescu H Levey DF Ayalew M Palmer L Gavrin LM Jain N Discovery and validation of blood biomarkers for suicidality Mol Psychiatry 2013 164 118 122 10.1038/mp.2013.95 PMC3835939 23958961

[bib10] Keri S Szabo C Kelemen O Blood biomarkers of depression track clinical changes during cognitive-behavioral therapy J Affect Disord 2014 164 118 122 10.1016/j.jad.2014.04.030 24856564

[bib11] Rice L Waters CE Eccles J Garside H Sommer P Kay P Identification and functional analysis of SKA2 interaction with the glucocorticoid receptor J Endocrinol 2008 198 499 509 10.1677/JOE-08-0019 PMC2518725 18583474

[bib12] Gillespie CF Bradley B Mercer K Smith AK Conneely K Gapen M Trauma exposure and stress-related disorders in inner city primary care patients Gen Hosp Psychiatry 2009 31 505 514 19892208 10.1016/j.genhosppsych.2009.05.003 PMC2785858

[bib13] Ressler KJ Mercer KB Bradley B Jovanovic T Mahan A Kerley K Post-traumatic stress disorder is associated with PACAP and the PAC1 receptor Nature 2011 470 492 497 21350482 10.1038/nature09856 PMC3046811

[bib14] Binder EB Bradley RG Liu W Epstein MP Deveau TC Mercer KB Association of FKBP5 polymorphisms and childhood abuse with risk of posttraumatic stress disorder symptoms in adults JAMA 2008 299 1291 1305 18349090 10.1001/jama.299.11.1291 PMC2441757

[bib15] Bradley RG Binder EB Epstein MP Tang Y Nair HP Liu W Influence of child abuse on adult depression: moderation by the corticotropin-releasing hormone receptor gene Arch Gen Psychiatry 2008 65 190 200 18250257 10.1001/archgenpsychiatry.2007.26 PMC2443704

[bib16] Kellam SG Werthamer-Larsson L Dolan LJ Brown CH Mayer LS Rebok GW Developmental epidemiologically based preventive trials: baseline modeling of early target behaviors and depressive symptoms Am J Commun Psychol 1991 19 563 584 10.1007/BF00937992 1755436

[bib17] Kellam SG Rebok GW Ialongo N Mayer LS The course and malleability of aggressive behavior from early first grade into middle school: results of a developmental epidemiologically-based preventive trial J Child Psychol Psychiatry 1994 35 259 281 8188798 10.1111/j.1469-7610.1994.tb01161.x

[bib18] Kellam SG Brown CH Poduska JM Ialongo NS Wang W Toyinbo P Effects of a universal classroom behavior management program in first and second grades on young adult behavioral, psychiatric, and social outcomes Drug Alcohol Depend 2008 95 S5 S28 18343607 10.1016/j.drugalcdep.2008.01.004 PMC2512256

[bib19] Mehta D Klengel T Conneely KN Smith AK Altmann A Pace TW Childhood maltreatment is associated with distinct genomic and epigenetic profiles in posttraumatic stress disorder Proc Natl Acad Sci USA 2013 110 8302 8307 23630272 10.1073/pnas.1217750110 PMC3657772

[bib20] Sun YV Smith AK Conneely KN Chang Q Li W Lazarus A Epigenomic association analysis identifies smoking-related DNA methylation sites in African Americans Hum Genet 2013 132 1027 1037 23657504 10.1007/s00439-013-1311-6 PMC3744600

[bib21] Thiede C Prange-Krex G Freiberg-Richter J Bornhauser M Ehninger G Buccal swabs but not mouthwash samples can be used to obtain pretransplant DNA fingerprints from recipients of allogeneic bone marrow transplants Bone Marrow Transplant 2000 25 575 577 10713640 10.1038/sj.bmt.1702170

[bib22] Smith AK Kilaru V Klengel T Mercer KB Bradley B Conneely KN DNA extracted from saliva for methylation studies of psychiatric traits: Evidence tissue specificity and relatedness to brain Am J Med Genet B Neuropsychiatr Genet 2015 168 36 44 25355443 10.1002/ajmg.b.32278 PMC4610814

[bib23] Smith AK Kilaru V Kocak M Almli LM Mercer KB Ressler KJ Methylation quantitative trait loci (meQTLs) are consistently detected across ancestry, developmental stage, and tissue type BMC Genomics 2014 15 145 24555763 10.1186/1471-2164-15-145 PMC4028873

[bib24] Coryell W Schlesser M The dexamethasone suppression test and suicide prediction Am J Psychiatry 2001 158 748 753 11329397 10.1176/appi.ajp.158.5.748

[bib25] McGirr A Diaconu G Berlim MT Pruessner JC Sable R Cabot S Dysregulation of the sympathetic nervous system, hypothalamic-pituitary-adrenal axis and executive function in individuals at risk for suicide J Psychiatry Neurosci 2010 35 399 408 20731961 10.1503/jpn.090121 PMC2964370

[bib26] Polanczyk G Caspi A Williams B Price TS Danese A Sugden K Protective effect of CRHR1 gene variants on the development of adult depression following childhood maltreatment: replication and extension Arch Gen Psychiatry 2009 66 978 985 19736354 10.1001/archgenpsychiatry.2009.114 PMC3750953

[bib27] Buchmann AF Holz N Boecker R Blomeyer D Rietschel M Witt SH Moderating role of FKBP5 genotype in the impact of childhood adversity on cortisol stress response during adulthood Eur Neuropsychopharmacol 2014 24 837 845 24411633 10.1016/j.euroneuro.2013.12.001

[bib28] Binder EB Holsboer F Low cortisol and risk and resilience to stress-related psychiatric disorders Biol Psychiatry 2012 71 282 283 22265026 10.1016/j.biopsych.2011.12.008

[bib29] Heim C Ehlert U Hellhammer DH The potential role of hypocortisolism in the pathophysiology of stress-related bodily disorders Psychoneuroendocrinology 2000 25 1 35 10633533 10.1016/s0306-4530(99)00035-9

[bib30] Kang JI Chung HC Jeung HC Kim SJ An SK Namkoong K FKBP5 polymorphisms as vulnerability to anxiety and depression in patients with advanced gastric cancer: a controlled and prospective study Psychoneuroendocrinology 2012 37 1569 1576 22459275 10.1016/j.psyneuen.2012.02.017

[bib31] Zimmermann P Bruckl T Nocon A Pfister H Binder EB Uhr M Interaction of FKBP5 gene variants and adverse life events in predicting depression onset: results from a 10-year prospective community study Am J Psychiatry 2011 168 1107 1116 21865530 10.1176/appi.ajp.2011.10111577 PMC3856576

