# Supplementary material for: Epigenetic and genetic variation at SKA2 predict suicidal behavior and post-traumatic stress disorder
Source: Transl Psychiatry. 2015 Aug 25;5(8):e627–. doi: 10.1038/tp.2015.105 (PMC4564560; doi:10.1038/tp.2015.105)
Supplement: Supplementary Information [file tp2015105x1.docx]

**Method S1**

Grady Trauma Project (GTP): The subjects for this study were part of a larger investigation of genetic and environmental factors that predict the response to stressful life events in a predominantly African American, urban population of low socioeconomic status (Gillespie et al., 2009).

Subjects were scored as having PTSD if they met DSM-IV criteria for lifetime PTSD based a structured interview ^1, 2^ (Clinician Administered PTSD Screen – CAPS – or the MINI International Neuropsychiatric Interview – M.I.N.I). Suicide attempt (SA) was measured as a binary response to the question: Have you ever tried to kill yourself or commit suicide? Current suicidal ideation (SI) was operationalized using item 9 of the Beck Depression Inventory (BDI), with SI being coded as present if the subject reports any of the following: 1) I have thoughts of harming myself but I would not carry them out, 2) I feel I would be better off dead, 3) I have definitive plans about committing suicide, 4) I feel my family would be better off if I were dead, or 5) I would kill myself if I could. Past history of substance abuse was captured by self report in response the question: Have you ever had a problem with drug or alcohol abuse? The Childhood Trauma Questionnaire (CTQ) was used to assess physical, sexual, and emotional abuse during childhood based on the established scores for mild, moderate, and severe abuse for each type ^3, 4^. All other lifetime trauma exposure was measured using the Traumatic Experiences Inventory (TEI) ^5^. Finally, anxiety symptoms were assessed as a continuous score from the Hamilton Anxiety Scale (HAM-A) ^6^.

All procedures were approved by the Institutional Review Board of Emory University School of Medicine and the Grady Health Systems Research Oversight Committee.

Johns Hopkins Center for Prevention Research Study (PRC): Data are from a prospective study conducted in the context of an epidemiologically-based group-randomized prevention trial ^7, 8^. Details of the trial are available elsewhere ^7, 8^.

All study metrics were derived as responses to a standardized interview ^7, 8^. Attrition in the cohort was slightly greater among males and whites (p<.01). Standardized assessments were conducted by trained non-clinical interviewers with the most recent wave collected via a computerized interview that was conducted by the interviewer, and when assessing potentially sensitive topics such as drug involvement, conducted by the respondent using the computer). A binary substance abuse metric was calculated such that individuals abusing or dependent on alcohol, tranquilizers, sedatives, marijuana, heroin, crack, cocaine, or other hallucinogens were coded as 1 and all those without reported abuse or dependence of any of these substances were coded as 0. Current suicidal ideation was determined at the interview closest to blood draw and measured as a binary response to the question: Have you ever felt so low you thought of committing suicide? Suicide attempt was measured as a binary response to the question: Have you ever attempted suicide? Childhood physical abuse that occurred prior to the age of 18 was assessed by self-report at 19-21 years of age. Subjects were asked "How often did your caregivers hit you hard enough to cause a bruise?" and "How often did your caregivers hit you hard enough to cause bleeding or break a bone?" Similarly, childhood emotional abuse was quantified by asking subjects “How often did your caregivers insult, swear, or yell at you?” and “How often did your caregivers threaten to end their relationships with you?”. Responses were numerically quantified from 1 to 6 corresponding to “Never, Rarely”, “Sometimes”, “Often”, “Most of the time”, and “Always”, respectively.  Childhood sexual trauma was assessed by self-report on a section pf the 1996 Detroit Area Survey ^9^. Sexual abuse was coded as a binary variable for individuals who reported sexual assault or rape prior to the age of 18. A single childhood trauma score was derived by taking the Eigen vector of the first principle component of the responses across emotional, physical, and sexual abuse domains.

This study was approved by the Institutional Review Board at Johns Hopkins University. All participants provided informed consent to participate.

**Method S2**

*SKA2* 3’UTR DNA methylation levels at rs7208505 were determined using normalized beta values for the cg13989295 probe from the Illumina HumanMethylation450 BeadChip from data generated previously ^10, 11^ in the GTP cohort. Beta values were generated with BeadStudio and were set to missing (no call) if detection p-values exceeded .001. CpGassoc ^12^ was used to remove samples with probe detection call rates <95% and those with an average intensity value of either <50% of the experiment-wide sample mean or <2,000 arbitrary units (AU). In addition, CpG sites with missing data for >10% of samples were excluded from analysis. Beta Mixture Quantile dilation (BMIQ) was used to normalize each dataset ^13^.

Genotyping was performed using the Omni-Quad 1M or the Omni Express BeadChip (Illumina). PLINK was used to perform quality control analyses such that SNPs that had a call rate < 95%, a minor allele frequency (MAF) < .05, or significant deviation from Hardy-Weinberg proportions (p < .00001) were excluded, as were samples with > 5% missing data. From this data, MaCH 1.0 ^14^ was used to impute GTP genotypes for rs7208505 using unrelated individuals from HapMap ASW, CEU, LWK, MKK, TSI, and YRI Phase 3 reference samples. Out of 61 of 421 subjects were missing genotype calls. Based on the close correlation between *SKA2* 3’UTR DNA methylation and rs7208505, we inferred the genotype from the remaining 61 samples by the following method. We built a linear discriminant analysis of called genotypes as a function of DNA methylation at rs7208505. DNA methylation values for the 61 samples with missing genotypes were input into the model, generating genotype call predictions that were subsequently used. This method returned the exact same values as calling genotype based on which sub distribution of the trimodal methylation distribution the individual’s methylation fell into (Figure S1).

The genotype, suicide attempt, and associated beta value distribution per genotype are as follows: TT homozygotes, N=231, beta range=0.04-0.18, mean=0.083 ± 0.021, suicide attempt N=53, non-attempt N=178; CT heterozygotes: N=174, beta range=0.49-0.69, mean=0.58 ± 0.029 suicide attempt N=43, non-attempt N= 31; CC homozygotes, N=16, beta range= 0.82-0.92, mean=0.89 ± 0.029 , suicide attempt N=3, non-attempt N=12.

**Method S3**

We assessed the effect that substance abuse, early life trauma and other potential confounding factors had on DNA methylation at rs7208505 using available information in the GTP cohort. After controlling for rs7208505 genotype, age, sex, only four factors demonstrated evidence for a significant effect on *SKA2* DNA methylation including the first two principle components of ancestry based on previously published GWA data ^15^ and past history of substance abuse (Table S2). Importantly, current substance abuse did not show a significant association (Table S2). Early life and adult trauma scores do not influence the levels of *SKA2* DNA methylation directly (Table S2); however, a further analysis demonstrates that both childhood trauma question are (CTQ) totals and TEI scale derived adult trauma exposure totals are significantly associated with past substance abuse (β=6.86 ± 1.85, F=13.65, df=1/405, p=2.5x10^-4^, and β=2.29 ± 0.3, F=57.01, df=1/402, p=2.9x10^-13^, respectively). *SKA2* DNA methylation demonstrated a continued association for post history of substance abuse when controlling for child abuse or lifetime trauma exposure as an additive covariate (Substance Abuse β= -0.0063 ± 0.0025, F= 3.28, df= 2/404, p=0.013 and Substance Abuse β = -0.0057 ± 0.0027, F=2.3, df= 2/401, p=0.034 respectively). For this reason, in order to accurately assess model predictions with trauma independent of substance related decreases on *SKA2* DNA methylation, we adjusted *SKA2* DNA methylation levels for past history of substance abuse in all analyses where we could not include substance abuse as a covariate in the regression.

**Result S1**

We performed the exploratory sliding window analysis on different subtypes of trauma reported in the CTQ including physical abuse, sexual abuse, and emotional abuse, assessing *SKA2* 3’UTR epigenetic and genetic prediction models on lifetime suicide attempt. The resulting plots demonstrate that emotional abuse most closely resembles the pattern observed for the total CTQ scores, while both physical and sexual abuse do not generate predictions for any windows where the 95% confidence limits do not include 0.5 (Figure S2).

**Result S2**

In the GTP cohort, the total anxiety score (HAM-A) is significantly associated with physical (β= 0.18 ± 0.084, F=5.02, df=1/347, p=0.026) and sexual (β=0.38 ± 0.11, F=12.94, df=1/348, p=3.7x10^-4^) abuse but is most strongly associated with emotional abuse (β=0.63 ± 0.089, F=50.27, df=1/347, p=7.5x10^-12^). The model performed significantly stronger in delineating 6 suicide attempters from 12 non-attempters experiencing severe emotional abuse but no to moderate physical or sexual abuse (AUC=0.81, 95% CI: 0.58-1, permuted p=0.066); however, the small sample size in this case resulted in only trend level significance and calls into question the usefulness of this refined prediction. Incorporation of CTQ emotional abuse scores as an interactive covariate improved model performance, generating a significant AUC of 0.86 (95% CI: 0.67-1, permuted p=0.028). This interactive model was also able to distinguish 5 PTSD cases from 9 non-PTSD cases who experienced severe emotional abuse but no to moderate physical or sexual trauma with an AUC of 0.84 (95% CI: 0.61-1, permuted p=0.066).

These findings should be interpreted in the context that there is often a higher prevalence of reported emotional relative to physical and sexual abuse in the population. The frequency of individuals categorized as experiencing severe physical and emotional abuse was similar at 26.6% and 25.6%, respectively, which is similar to the 24% reported by young adults in larger epidemiological surveys ^16^. The frequency of severe sexual abuse reported in the GTP was higher at 34.4%. These findings suggest the better performance of the emotional abuse metric in the model may not be driven by a larger portion of the cohort reporting this form of abuse and should be investigated further in future studies.

**Result S3**

We assessed other CpGs within the *SKA2* locus for association with either suicide or PTSD (Table S4). Many CpGs within the *SKA2* gene were significantly correlated with rs7208505 genotype adjusted DNA methylation after Bonferroni correction (Table S4). No associations were observed with suicide attempt in a linear model correcting for age, sex, race, and past substance abuse. By contrast, three CpGs located in the *SKA2* promoter region exhibited nominally significant association to PTSD that was not significant after correction for multiple testing. Gene expression decreases at *SKA2* have been associated with suicidal behaviors and completed suicide ^17, 18^. Our previous work identified a three way interaction between *SKA2* 3’UTR DNA methylation and that of microRNA 301a (miR-301a) (cg10822495) and three promoter CpGs proximal to a CREB1 binding site that drives *SKA2* transcription in conjunction with miR-301a modulation ("cg01515809", "cg20009499","cg17663700") ^17^. Similar to our previous study, we observed significant correlations between rs7208505 adjusted DNA methylation (cg13989295) and the CpGs in the miR-301a (Rho= 0.23, p= 1.1x10^-6^) and promoter region (Rho= -0.19, p= 9.8x10^-5^).

Peripheral blood gene expression levels for *SKA2* derived from Illumina expression microarray probe (downloaded from http://www.ncbi.nlm.nih.gov/geo/ Accession # GSE42002), ILMN_1807807, were not associated with suicide attempt or PTSD in this cohort (Wilcoxon Rank Sum Test Suicide: N= 99, Mean= 6.5 + 0.18, non-Suicide: N= 321, Mean= 6.5 + 0.17, p= 0.29: PTSD: N= 78, Mean= 6.5 + 0.17, non-PTSD: N= 203, Mean= 6.5 + 0.17, p= 0.79). We did observe a significant replication of the three-way interaction between *SKA2* 3’UTR, miR-301a, and CREB1 proximal CpG DNA methylation (Table S5). Similar results were obtained when using the average of all promoter CpGs (data not shown). Incorporation of the miR-301a and promoter CpGs into the interactive model with CTQ scores, correcting for age, sex, race, and past substance abuse improved the model R2 to 0.32 from 0.25 (Table 2, Table S5). No significant interactions were observed for this model with PTSD (data not shown); however, *SKA2* promoter DNA methylation was independently associated with PTSD after controlling for CTQ, age, sex, race, and substance abuse history (b= 7.67 + 3.6, F= 7.9, df=8/261, p= 0.034). Cumulatively, the data suggest that epigenetic variation in other gene expression relevant CpG regions of *SKA2* may be important for modeling disease associated variation and should be investigated further in future studies.


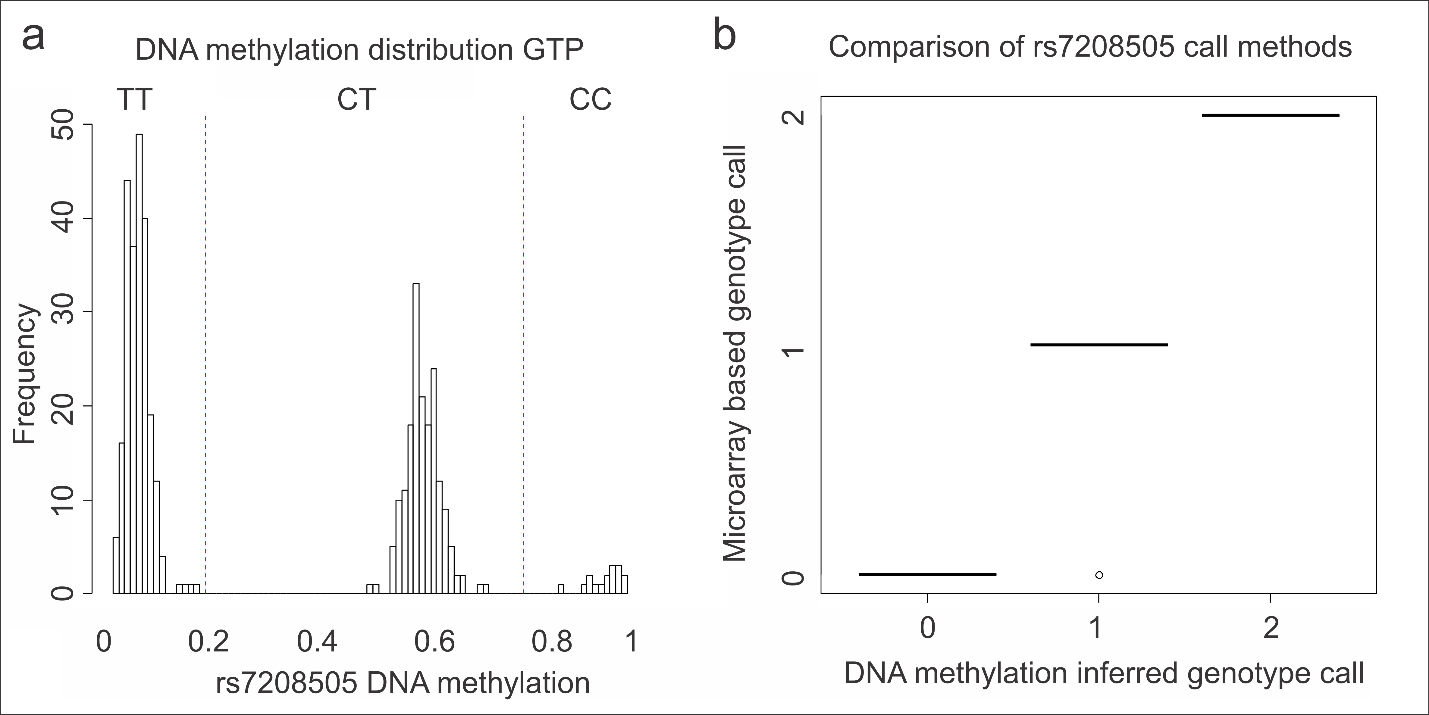


**Figure S1. Genotyping of rs7208505 from DNA methylation data**

a.) A histogram of DNA methylation at rs7208505 in the GTP cohort enabling genotype calling of N=61 subjects with missing genotype data. Dashed vertical red lines denote cut offs segregating DNA methylation inferred genotype calls using linear discriminant analysis. b.) A boxplot of genotype calls based on microarray genotyping (y axis) as a function of genotype calls inferred by the linear discriminate analysis of genotype based on DNA methylation or DNA methylation distribution. Out of the N=360 subjects with both measures, a single outlier was detected.


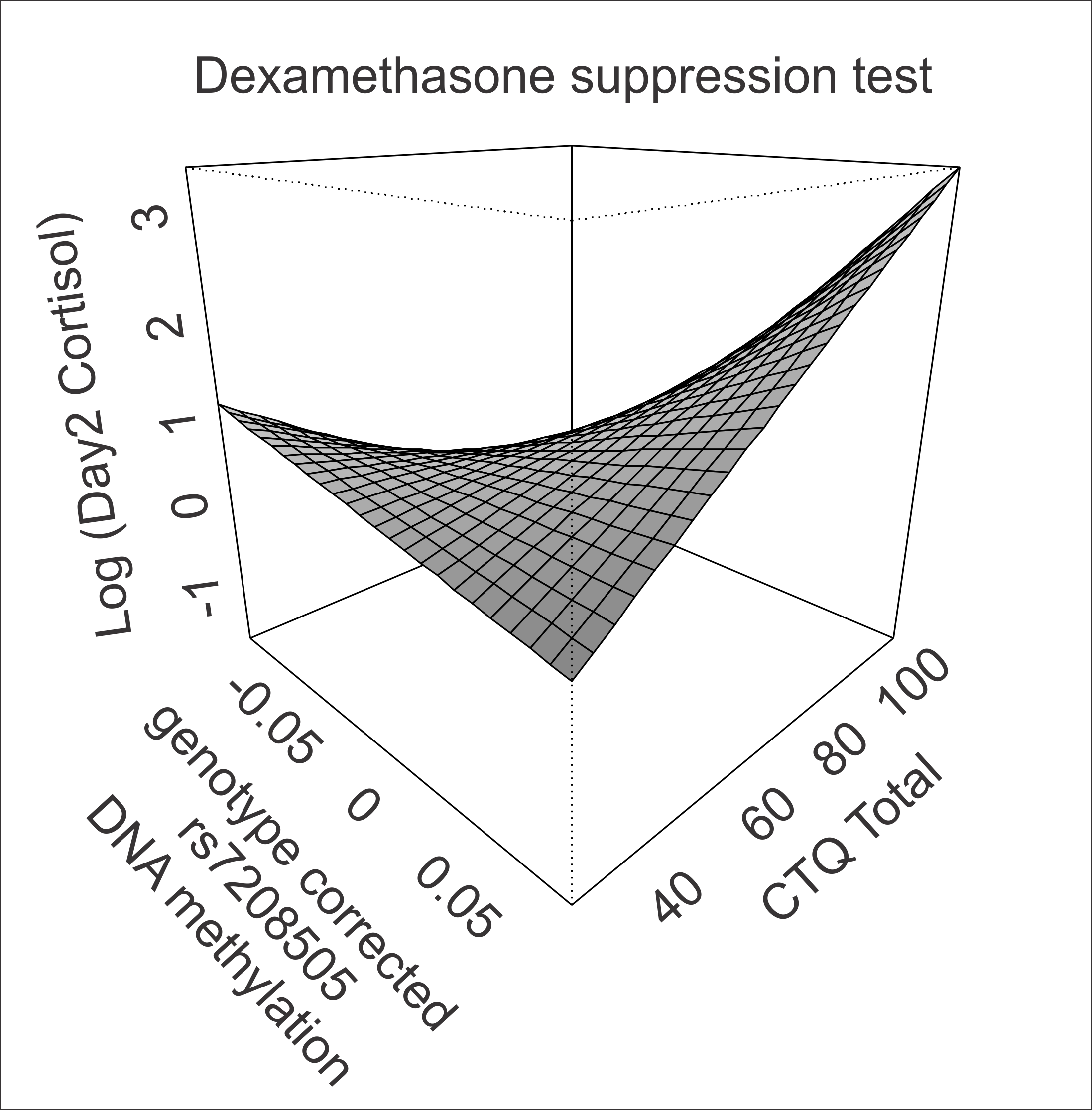


**Figure S2. *SKA2* moderation of trauma on post DST cortisol levels**

A three dimensional representation of the significant model of rs7208505 corrected *SKA2* 3’UTR DNA methylation (x axis) interacting with CTQ trauma scores (z axis) and its effect on the natural log of the day 2 cortisol values following the dexamethasone suppression test (y axis).

| **Table S1:** *Clinical and Demographic Characteristics of the Cohorts* | | | |
| --- | --- | --- | --- |
|  | **GTP Blood, N=421** | **GTP Saliva, N=61** | **PRC Blood, N=325** |
| Age, mean ± SD | 41.6 ± 12.7 | 42.4 ± 11.8 | 30.4 ± 2.5 |
| Female Sex, N (%) | 299 (71.0%) | 48 (78.7%) | 197 (60.6%) |
| African American Race, N (%) | 392 (93.1%) | 54 (88.5%) | 247 (76%) |
| SA Lifetime, N (%) | 99 (23.6%) | 19 (31.1%) | 48 (14.8%) |
| SI Current, N (%) | 67 (16.6%) | 8 (13.8%) | 78 (24%) |
| Current PTSD, N (%) | 78 (27.7%) |  |  |
| CTQ Total, mean ± SD | 43.7 ± 18.4 | 48.7 ± 23.4 | 131 (40.3%)* |
| Past Substance Abuse, N (%) | 151 (37.4%) | 24 (39.3%) | 96 (29.5%) |
| HAM-A Score, mean ± SD | 11.1 ± 8.9 | 12.6 ± 9.7 |  |

* Number (%) of individuals in the PRC cohort were classified as traumatized based on reported physical or sexual violence exposure.

| **Table S2:** *Confounding influences on SKA2 DNA methylation* | | | | | |  |  |  |  |  |
| --- | --- | --- | --- | --- | --- | --- | --- | --- | --- | --- |
| **DNA methylation association with substance/diagnosis** | | |  |  |  | **Suicide association with substance/diagnosis** | | | |  |
| **Covariate** | **β value** | **Error** | **F** | **DF** | **Model P** | **β value** | **Error** | **F** | **DF** | **Model P** |
| Ancestry PC2 | 0.76 | 0.24 | 10 | 266 | 0.0016 | 8.2 | 4.1 | 4.1 | 266 | 0.044 |
| Ancestry PC1 | -0.25 | 0.096 | 6.6 | 266 | 0.011 | -2.3 | 1.6 | 2 | 266 | 0.15 |
| Lifetime Substance Abuse | -0.006 | 0.0025 | 5.6 | 413 | 0.019 | 0.27 | 0.041 | 42 | 413 | 2.5x10^-10^ |
| Current Substance Abuse | -0.008 | 0.0053 | 2 | 409 | 0.16 | 0.12 | 0.091 | 1.7 | 409 | 0.19 |
| Lifetime Bipolar | -0.007 | 0.0061 | 1.5 | 357 | 0.23 | 0.27 | 0.1 | 7 | 356 | 0.0085 |
| Smoker Life | 0.001 | 0.0038 | 0.075 | 229 | 0.78 | 0.087 | 0.06 | 2.1 | 229 | 0.15 |
| Lifetime MDD | 0.0004 | 0.0026 | 0.027 | 354 | 0.87 | 0.15 | 0.043 | 12 | 353 | 0.0008 |
| Current MDD | 0.0005 | 0.0036 | 0.017 | 340 | 0.9 | 0.2 | 0.059 | 11 | 336 | 0.0009 |
| Current Bipolar | -1E-03 | 0.0084 | 0.013 | 357 | 0.91 | 0.0073 | 0.14 | 0.0028 | 356 | 0.96 |

| **Table S3:** *Trauma subtype interactive effects on suicide phenotypes* | | | |
| --- | --- | --- | --- |
| **Sample** |  | **PRC Suicidal Attempt(N=325)** |  |
| **Model Terms** | **β value** | **Error** | **P value** |
| DNAm | -0.01 | 0.0028 | 0.02 |
| C/T | 0.31 | 0.15 | 0.03 |
| C/C | 0.45 | 0.24 | 0.07 |
| Trauma | 0.04 | 0.03 | 0.21 |
| Age | -0.01 | 0.01 | 0.38 |
| Sex | 0.0027 | 0.04 | 0.95 |
| Past Substance Abuse | 0.15 | 0.04 | 0.0003 |
| DNAm × Emotional Abuse | 0.0036 | 0.0014 | 0.01 |
| C/T × Emotional Abuse | -0.14 | 0.07 | 0.05 |
| C/C × Emotional Abuse | -0.27 | 0.13 | 0.03 |
| F | 3 |  |  |
| DF | 12/257 |  |  |
| Model R^2^ | 0.12 |  | 5.91x10^-4^ |
| DNAm | -0.0024 | 0.0025 | 0.33 |
| C/T | 0.23 | 0.13 | 0.09 |
| C/C | 0.37 | 0.25 | 0.14 |
| Trauma | 0.06 | 0.04 | 0.17 |
| Age | -0.01 | 0.01 | 0.44 |
| Sex | 0.01 | 0.05 | 0.83 |
| Past Substance Abuse | 0.15 | 0.041 | 0.0002 |
| DNAm × Physical Abuse | 0.0019 | 0.0019 | 0.32 |
| C/T × Physical Abuse | -0.15 | 0.10 | 0.13 |
| C/C × Physical Abuse | -0.34 | 0.20 | 0.09 |
| F | 2.49 |  |  |
| DF | 12/257 |  |  |
| Model R^2^ | 0.1 |  | 0.0043 |
| DNAm | 0.000 | 0.001 | 0.89 |
| C/T | 0.009 | 0.054 | 0.87 |
| C/C | 0.025 | 0.090 | 0.78 |
| Trauma | 0.28 | 0.082 | 0.001 |
| Age | -0.001 | 0.01 | 0.89 |
| Sex | 0.000 | 0.04 | 1.00 |
| Past Substance Abuse | 0.11 | 0.036 | 0.003 |
| DNAm × Sexual Abuse | 0.002 | 0.003 | 0.46 |
| C/T × Sexual Abuse | -0.03 | 0.15 | 0.84 |
| C/C × Sexual Abuse | -0.37 | 0.23 | 0.11 |
| F | 3.6 |  |  |
| DF | 12/257 |  |  |
| Model R^2^ | 0.13 |  | 2.66x10^-5^ |

| **Table S4:** *Association of SKA2 CpG probes with suicide attempt (SA), PTSD, and SKA2 3’UTR methylation* | | | | | | | | | |
| --- | --- | --- | --- | --- | --- | --- | --- | --- | --- |
|  |  | **SA Association** | | | **PTSD Association** | | | ***SKA2* 3'UTR^+^**  **Association** | |
| **Probe ID** | **Chr 17 Position** | **β value** | **Error** | **P value** | **β value** | **Error** | **P value** | **Rho** | **P value*** |
| cg01964121 | 54571258 | 9.11 | 12.55 | 0.47 | 18.06 | 15.48 | 0.24 | 0.20 | 6.01x10^-4^ |
| cg10325038 | 54571266 | 0.03 | 0.63 | 0.96 | 1.25 | 0.79 | 0.11 | 0.20 | 5.82 x10^-4^ |
| cg10822495 | 54583355 | -0.02 | 0.54 | 0.97 | -0.09 | 0.72 | 0.90 | 0.26 | 1.94 x10^-6^ |
| cg02656609 | 54583400 | -0.48 | 1.37 | 0.73 | -2.27 | 1.93 | 0.24 | 0.22 | 8.72 x10^-5^ |
| cg07505964 | 54584296 | 0.05 | 1.01 | 0.96 | 0.30 | 1.46 | 0.84 | 0.24 | 1.68 x10^-5^ |
| cg19273756 | 54584473 | 0.04 | 0.71 | 0.95 | 0.38 | 1.00 | 0.71 | 0.16 | 0.02 |
| cg12169852 | 54586508 | 2.24 | 3.34 | 0.50 | 0.64 | 4.43 | 0.88 | -0.10 | 1 |
| cg07333037 | 54586638 | 1.37 | 2.47 | 0.58 | -5.08 | 3.33 | 0.13 | -0.17 | 6.95 x10^-3^ |
| cg09972077 | 54587073 | 0.80 | 1.56 | 0.61 | -1.62 | 2.08 | 0.44 | -0.16 | 0.01 |
| cg01515809 | 54587178 | 0.69 | 1.25 | 0.58 | -3.59 | 1.66 | 0.031 | -0.17 | 0.01 |
| cg20009499 | 54587349 | 1.02 | 4.66 | 0.83 | -1.47 | 6.33 | 0.82 | -0.15 | 0.04 |
| cg17663700 | 54587356 | -2.49 | 3.47 | 0.47 | -0.96 | 4.87 | 0.84 | -0.17 | 0.01 |
| cg17989037 | 54587366 | -9.31 | 11.19 | 0.41 | -31.82 | 16.02 | 0.048 | -0.13 | 0.19 |
| cg27512082 | 54587671 | -0.27 | 4.55 | 0.95 | -2.86 | 6.28 | 0.65 | -0.21 | 2.68 x10^-4^ |
| cg12941374 | 54587743 | 1.08 | 4.02 | 0.79 | -9.89 | 5.11 | 0.054 | -0.11 | 0.40 |
| cg16861410 | 54587795 | 0.14 | 5.39 | 0.98 | -0.63 | 8.34 | 0.94 | -0.16 | 1.77 x10^-2^ |
| cg02573089 | 54587824 | -1.23 | 2.98 | 0.68 | 1.57 | 3.97 | 0.69 | -0.07 | 1 |
| cg19178362 | 54587829 | -1.70 | 2.64 | 0.52 | 1.68 | 3.50 | 0.63 | -0.19 | 2.04 x10^-3^ |
| cg09726208 | 54587840 | 1.59 | 2.35 | 0.50 | -1.76 | 3.21 | 0.58 | -0.14 | 0.09 |
| cg11214846 | 54587848 | -0.42 | 2.71 | 0.88 | 0.37 | 3.73 | 0.92 | -0.12 | 0.32 |
| cg24616461 | 54587897 | -1.28 | 2.16 | 0.55 | -4.62 | 2.93 | 0.12 | -0.26 | 1.51 x10^-6^ |

***=** Bonferroni corrected

**^+^** = methylation levels of cg13989295 adjusted for rs7208505 genotype

| **Table S5:**  *Interaction of additional SKA2 CpGs* | | |  |
| --- | --- | --- | --- |
|  |  | **SKA2 Gene Expression** |  |
| **Model Term** | **β value** | **Error** | **P value** |
| 3'UTR DNAm | -36.14 | 17.86 | 0.044 |
| miR-301a | 17.41 | 9.41 | 0.065 |
| Promoter | 30.36 | 14.74 | 0.040 |
| rs7208505 C/T | 7.99 | 4.46 | 0.074 |
| rs7208505 C/C | 412.16 | 249.66 | 0.100 |
| 3'UTR DNAm X miR-301a | 39.82 | 19.05 | 0.037 |
| rs7208505 C/T X miR-301a | -19.24 | 10.01 | 0.055 |
| rs7208505 C/C X miR-301a | -33.42 | 15.64 | 0.033 |
| 3'UTR DNAm X Promoter | 2114.15 | 1043.75 | 0.044 |
| rs7208505 C/T X Promoter | -1030.88 | 544.90 | 0.059 |
| rs7208505 C/C X Promoter | -1775.05 | 873.11 | 0.043 |
| miR-301a X Promoter | -456.64 | 267.24 | 0.088 |
| 3'UTR DNAm X miR-301a X Promoter | -2327.62 | 1120.33 | 0.039 |
| rs7208505 C/T X miR-301a X Promoter | 1137.94 | 583.74 | 0.052 |
| rs7208505 C/C X miR-301a X Promoter | 1951.51 | 933.69 | 0.037 |
| F | 0.98 |  |  |
| DF | 15/341 |  |  |
| Model R^2^ | 0.4 |  | 0.45 |
|  |  | **Suicide Attempt** |  |
| **Model Term** | **β value** | **Error** | **P value** |
| 3'UTR DNAm | 186.80 | 112.10 | 0.10 |
| rs7208505 C/T | -94.71 | 57.07 | 0.10 |
| rs7208505 C/C | -95.25 | 106.80 | 0.37 |
| miR-301a | -45.87 | 28.14 | 0.10 |
| Promoter | -2565.00 | 1418.00 | 0.071 |
| CTQ | -0.89 | 0.65 | 0.17 |
| Age | 0.00 | 0.00 | 0.90 |
| Sex | 0.12 | 0.04 | 0.0039 |
| Race1 | 0.08 | 0.09 | 0.34 |
| Race2 | 0.63 | 0.24 | 0.010 |
| Race3 | 0.03 | 0.37 | 0.93 |
| Substance Abuse History | 0.16 | 0.05 | 0.0007 |
| 3'UTR DNAm X miR-301a | -217.00 | 120.90 | 0.074 |
| rs7208505 C/T X miR-301a | 110.00 | 61.51 | 0.075 |
| rs7208505 C/C X miR-301a | 116.50 | 114.20 | 0.31 |
| 3'UTR DNAm X Promoter | -12660.00 | 6052.00 | 0.037 |
| rs7208505 C/T X Promoter | 6155.00 | 3081.00 | 0.047 |
| rs7208505 C/C X Promoter | 8633.00 | 6116.00 | 0.16 |
| miR-301a X Promoter | 2928.00 | 1529.00 | 0.056 |
| 3'UTR DNAm X CTQ | -4.12 | 2.80 | 0.14 |
| rs7208505 C/T X CTQ | 2.08 | 1.39 | 0.13 |
| rs7208505 C/C X CTQ | 1.46 | 3.00 | 0.63 |
| miR-301a X CTQ | 1.05 | 0.69 | 0.13 |
| Promoter X CTQ | 63.37 | 33.41 | 0.059 |
| 3'UTR DNAm X miR-301a X Promoter | 14330.00 | 6529.00 | 0.029 |
| rs7208505 C/T X miR-301a X Promoter | -6991.00 | 3325.00 | 0.036 |
| rs7208505 C/C X miR-301a X Promoter | -9880.00 | 6549.00 | 0.13 |
| 3'UTR DNAm X miR-301a X CTQ | 4.80 | 2.99 | 0.11 |
| rs7208505 C/T X miR-301a X CTQ | -2.43 | 1.48 | 0.10 |
| rs7208505 C/C X miR-301a X CTQ | -1.90 | 3.19 | 0.55 |
| 3'UTR DNAm X Promoter X CTQ | 301.60 | 144.40 | 0.037 |
| rs7208505 C/T X Promoter X CTQ | -145.70 | 72.06 | 0.044 |
| rs7208505 C/C X Promoter X CTQ | -180.60 | 173.40 | 0.30 |
| miR-301a X Promoter X CTQ | -71.47 | 35.79 | 0.047 |
| 3'UTR DNAm X miR-301a X Promoter X CTQ | -338.10 | 154.80 | 0.030 |
| rs7208505 C/T X miR-301a X Promoter X CTQ | 164.10 | 77.34 | 0.035 |
| rs7208505 C/C X miR-301a X Promoter X CTQ | 206.10 | 184.70 | 0.27 |
| F | 4.68 |  |  |
| DF | 37/369 |  |  |
| Model R^2^ | 31.94 |  | 2.8x10^-15^ |

Supplementary References

1. Sheehan DV, Lecrubier Y, Sheehan KH, Amorim P, Janavs J, Weiller E *et al.* The Mini-International Neuropsychiatric Interview (M.I.N.I.): the development and validation of a structured diagnostic psychiatric interview for DSM-IV and ICD-10. *J Clin Psychiatry* 1998; **59 Suppl 20:** 22-33;quiz 34-57.

2. Blake DD, Weathers FW, Nagy LM, Kaloupek DG, Gusman FD, Charney DS *et al.* The development of a Clinician-Administered PTSD Scale. *Journal of traumatic stress* 1995; **8**(1)**:** 75-90.

3. Bernstein DP, Stein JA, Newcomb MD, Walker E, Pogge D, Ahluvalia T *et al.* Development and validation of a brief screening version of the Childhood Trauma Questionnaire. *Child abuse & neglect* 2003; **27**(2)**:** 169-190.

4. Fink LA, Bernstein D, Handelsman L, Foote J, Lovejoy M. Initial reliability and validity of the childhood trauma interview: a new multidimensional measure of childhood interpersonal trauma. *The American journal of psychiatry* 1995; **152**(9)**:** 1329-1335.

5. Sprang G. The traumatic experiences inventory (TEI): A test of psychometric properties. *Journal of Psychopathology and Behavioral Assessment* 1997; **19**(3)**:** 257-271

6. Maier W, Buller R, Philipp M, Heuser I. The Hamilton Anxiety Scale: reliability, validity and sensitivity to change in anxiety and depressive disorders. *J Affect Disord* 1988; **14**(1)**:** 61-68.

7. Kellam SG, Werthamer-Larsson L, Dolan LJ, Brown CH, Mayer LS, Rebok GW *et al.* Developmental epidemiologically based preventive trials: baseline modeling of early target behaviors and depressive symptoms. *Am J Community Psychol* 1991; **19**(4)**:** 563-584.

8. Kellam SG, Rebok GW, Ialongo N, Mayer LS. The course and malleability of aggressive behavior from early first grade into middle school: results of a developmental epidemiologically-based preventive trial. *J Child Psychol Psychiatry* 1994; **35**(2)**:** 259-281.

9. Breslau N, Kessler RC, Chilcoat HD, Schultz LR, Davis GC, Andreski P. Trauma and posttraumatic stress disorder in the community: the 1996 Detroit Area Survey of Trauma. *Arch Gen Psychiatry* 1998; **55**(7)**:** 626-632.

10. Mehta D, Klengel T, Conneely KN, Smith AK, Altmann A, Pace TW *et al.* Childhood maltreatment is associated with distinct genomic and epigenetic profiles in posttraumatic stress disorder. *Proceedings of the National Academy of Sciences of the United States of America* 2013; **110**(20)**:** 8302-8307.

11. Sun YV, Smith AK, Conneely KN, Chang Q, Li W, Lazarus A *et al.* Epigenomic association analysis identifies smoking-related DNA methylation sites in African Americans. *Hum Genet* 2013; **132**(9)**:** 1027-1037.

12. Barfield RT, Kilaru V, Smith AK, Conneely KN. CpGassoc: an R function for analysis of DNA methylation microarray data. *Bioinformatics* 2012; **28**(9)**:** 1280-1281.

13. Teschendorff AE, Marabita F, Lechner M, Bartlett T, Tegner J, Gomez-Cabrero D *et al.* A beta-mixture quantile normalization method for correcting probe design bias in Illumina Infinium 450 k DNA methylation data. *Bioinformatics* 2013; **29**(2)**:** 189-196.

14. Li Y, Willer CJ, Ding J, Scheet P, Abecasis GR. MaCH: using sequence and genotype data to estimate haplotypes and unobserved genotypes. *Genet Epidemiol* 2010; **34**(8)**:** 816-834.

15. Almli LM, Duncan R, Feng H, Ghosh D, Binder EB, Bradley B *et al.* Correcting systematic inflation in genetic association tests that consider interaction effects: application to a genome-wide association study of posttraumatic stress disorder. *JAMA psychiatry* 2014; **71**(12)**:** 1392-1399.

16. Radford L, Corral S, Bradley C, Fisher HL. The prevalence and impact of child maltreatment and other types of victimization in the UK: findings from a population survey of caregivers, children and young people and young adults. *Child abuse & neglect* 2013; **37**(10)**:** 801-813.

17. Guintivano J, Brown T, Newcomer A, Jones M, Cox O, Maher BS *et al.* Identification and replication of a combined epigenetic and genetic biomarker predicting suicide and suicidal behaviors. *The American journal of psychiatry* 2014; **171**(12)**:** 1287-1296.

18. Niculescu AB, Levey D, Le-Niculescu H, Niculescu E, Kurian SM, Salomon D. Psychiatric blood biomarkers: avoiding jumping to premature negative or positive conclusions. *Molecular psychiatry* 2015; **20**(3)**:** 286-288.
